# Learning Adaptive Search with Reinforcement Learning for Small and Fast Object Tracking

**DOI:** 10.3390/s26185819

**Published:** 2026-09-14

**Authors:** Binrui Liu, Xinyi Bo, Wenbin Luo, Haolun Li, Jingqi Wang, Ge Zheng, Shuiwang Li

**Affiliations:** 1 College of Computer Science and Engineering, Guilin University of Technology, Guilin 541006, China; 3232052051388@glut.edu.cn (B.L.); bxy51631@gmail.com (X.B.); 2120251305@glut.edu.cn (H.L.); wangjingqi@glut.edu.cn (J.W.); zhengge0729@glut.edu.cn (G.Z.); 2 Guangxi Science and Technology Information Network Center, Nanning 530022, China; luowenbin@kjt.gxzf.gov.cn

**Keywords:** visual tracking, small object tracking, reinforcement learning, adaptive input control, segmentation-based tracking

## Abstract

The persistent challenge in visual object tracking, particularly for small and fast-moving targets, lies in the trade-off between effective resolution and contextual information. Fixed search regions cannot adapt to variations in target scale and motion, often resulting in degraded target representation and tracking failures. In this paper, we introduce AdaSAM2, a framework that formulates adaptive search-region selection as a sequential decision-making problem. Unlike conventional per-frame heuristics, our approach employs an event-driven reinforcement learning policy that selects the cropping scale only during initialization and unreliable tracking states, while reusing the previous configuration during stable tracking. To handle target disappearance, we further introduce a lost-aware recovery mechanism that combines progressive search-region enlargement with constrained policy re-selection. Extensive experiments on TSFMO, LaTOT, UAV123, and UAVDT demonstrate consistent improvements over the SAMITE baseline. For example, AdaSAM2 improves Success and Precision on TSFMO from 42.6% and 74.0% to 43.9% and 75.3%, respectively, while improving UAV123 Success and Precision from 69.6% and 92.7% to 71.1% and 94.8%. Moreover, the RL policy is activated on only 0.74% of processed frames on TSFMO, resulting in an average overhead of only 0.0085 ms per frame. These results demonstrate that adaptive input-space optimization can improve tracking accuracy while introducing negligible computational overhead.

## 1. Introduction

In recent years, visual object tracking has achieved significant progress, demonstrating strong performance on standard benchmarks such as UAV123 [1] and UAVDT [2]. This progress has been driven by diverse advances in tracking architectures, including transformer-based trackers [3,4,5,6] that leverage object-centric modeling and global attention mechanisms, as well as emerging bio-inspired approaches based on spiking neural networks [7], which explore alternative computational paradigms for efficient visual tracking. Specifically, these methods are capable of modeling long-range dependencies and integrating spatial and temporal information, thereby enabling more accurate and stable target localization across frames. As a result, they have continuously advanced the performance of generic object tracking. Nevertheless, their remarkable success is primarily achieved on medium-to-large targets, while tracking extremely small objects remains considerably more challenging.

Representative small-object tracking benchmarks, such as TSFMO [8] and LaTOT [9], show that tiny targets, often occupying only a few pixels, are highly susceptible to large motion, motion blur, and drastic appearance changes, which leads to unstable tracking. In such cases, limited visual information severely weakens the discriminative capability of feature representations, while large inter-frame displacement further increases the uncertainty of target localization. Consequently, even advanced trackers are prone to drift or complete target loss [8,9,10]. This issue essentially reflects an inherent trade-off between resolution and context: methods focusing on local regions can enhance fine-grained details but often lack global contextual information, whereas methods relying on global modeling preserve context but struggle to effectively capture small targets [10].

**Figure 1:**
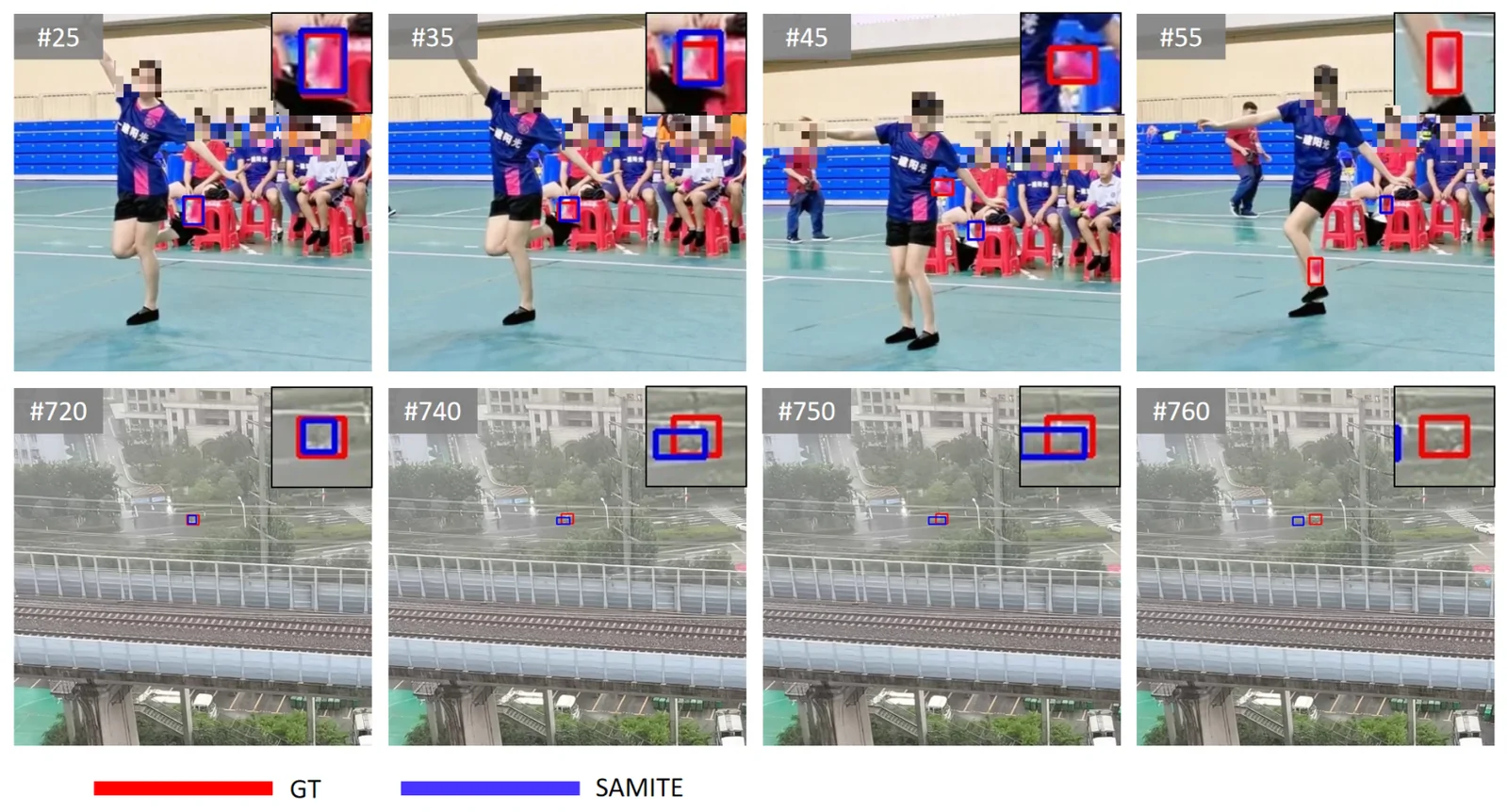
Failure cases of the SAMITE tracker under fast motion and blurry small-object scenarios. The tracker fails to accurately localize the target in these challenging conditions. Red boxes denote the ground truth, while blue boxes represent the predictions of SAMITE.

Recently, segmentation-based tracking methods built upon large-scale foundation models, such as SAM2 [11], SAMURAI [12], and the more recent SAMITE [13], have demonstrated strong generalization across diverse tracking scenarios. These methods establish a new tracking-by-segmentation paradigm by leveraging promptable segmentation and powerful pretrained representations. Among them, SAMITE further enhances the original SAM2 framework through calibrated memory and position prompting, providing a strong baseline for segmentation-based tracking. Nevertheless, their performance remains limited in small-object scenarios. Our analysis reveals that many tracking failures are not caused by insufficient model capacity, but rather by the inadequate effective resolution of tiny targets within the input image. As illustrated in Figure 1, when the target occupies only a small portion of the search region, the segmentation model often fails to generate reliable predictions. Reducing the search region can effectively increase the relative target resolution and significantly improve tracking quality. This observation suggests that input-space selection plays a critical role in balancing target discriminability and contextual information. However, existing SAM-based trackers generally employ fixed or heuristic search regions, which cannot adapt to rapid changes in target scale, motion patterns, or temporary tracking failures. Consequently, they often fail to maintain an appropriate resolution–context trade-off throughout the tracking process, motivating the need for an adaptive input-space optimization strategy.

To address these issues, we propose AdaSAM2, an adaptive input-space optimization framework that formulates search-region selection as a sequential decision-making problem and dynamically adjusts the search region according to the current tracking state. Therefore, we employ a reinforcement learning (RL)-based policy [14,15] to adaptively select the search region in a data-driven manner. This formulation enables the policy to explicitly optimize the resolution–context trade-off during inference. In addition, we introduce a lost-aware recovery mechanism that progressively enlarges the search region during recovery and periodically re-selects the cropping policy when unreliable tracking is detected, thereby mitigating error accumulation caused by inaccurate scale estimation. Furthermore, we design a multi-score fusion strategy that jointly considers appearance similarity, spatial consistency, and scale variation to produce more reliable target estimation. Unlike heuristic search-region adjustment strategies, the proposed policy explicitly learns the resolution–context trade-off from data, allowing adaptive cropping decisions under diverse tracking conditions. Overall, AdaSAM2 effectively increases target resolution while preserving sufficient contextual information, leading to consistent performance gains on both small-object and generic tracking benchmarks. The contributions of this paper are summarized as follows:

- We propose AdaSAM2, an event-driven adaptive search-region optimization framework that explicitly addresses the resolution–context trade-off in SAM2-based tracking. Search-region selection is formulated as an MDP, where a state-aware RL policy dynamically determines the appropriate search-region scale according to the tracking state.
- We design a lost-aware recovery mechanism that integrates progressive search-region enlargement with constrained policy re-selection, enabling stable recovery from target disappearance while preventing abrupt search-region changes.
- Extensive experiments on both tiny-object and general tracking benchmarks demonstrate that AdaSAM2 consistently improves tracking accuracy over the SAMITE baseline while maintaining high computational efficiency, validating the effectiveness and practicality of the proposed event-driven adaptive search-region optimization framework.

## 2. Relate Work

### 2.1. Small Object Tracking

Early tracking methods are mostly based on Siamese networks [16,17], formulating the tracking task as template matching; recent approaches adopt Transformer-based multi-type modeling [18,19] to improve tracking robustness via long-range dependencies. Despite these advances, small object tracking remains highly challenging due to limited visual information and susceptibility to background clutter, fast motion, and occlusion [10]. Existing methods enhance feature representations through multi-scale or hierarchical designs [20], yet these improvements primarily operate within the feature space and do not address the fundamental issue of insufficient effective resolution. This phenomenon reflects an inherent trade-off between resolution and context: local-focused methods improve discriminability but lose global context, whereas global modeling preserves contextual information yet often performs poorly on small targets. Some studies attempt to alleviate this issue through spatial-aware or context-aware mechanisms [17,21]. However, these strategies are generally heuristic and remain incapable of adaptively adjusting the input space according to changing tracking conditions. This limitation becomes even more pronounced in segmentation-based tracking, where the quality of the predicted mask is directly affected by the effective input resolution.

### 2.2. SAM-Based Methods

Recent advances in segmentation-based tracking have been driven by foundation models such as SAM [22] and its video extension SAM2 [11], and subsequent works [12,13,23] have further improved tracking performance by incorporating memory mechanisms. However, existing SAM-based trackers primarily focus on improving mask quality through enhanced memory mechanisms and decoder prompting strategies, as exemplified by SAMITE [13] and SAMURAI [12], while largely overlooking adaptive input-space optimization for improving effective target resolution. More importantly, the aforementioned methods generally lack adaptive input space control, making it difficult to effectively balance the trade-off between resolution enhancement and contextual preservation under dynamic conditions.

### 2.3. Reinforcement Learning for Tracking

Reinforcement learning typically formulates tasks as sequential decision-making problems, with representative frameworks including DQN [15] and PPO [24]. In early work, ADNet [25] treats tracking as an iterative action execution process, and subsequent methods further learn adaptive policies for tracking decisions [26,27], applying them to aspects such as strategy selection, template selection, and search region or scale estimation. However, existing approaches are predominantly based on bounding box representations and focus primarily on target state prediction or module selection [28]; in contrast, utilizing reinforcement learning to control input representations—particularly for dynamic search region adjustment in segmentation-based tracking tasks—remains largely unexplored. Existing RL-based trackers mainly optimize tracking decisions after feature extraction, whereas our method formulates adaptive input-space optimization as a sequential decision-making problem before feature extraction, providing a complementary perspective for improving segmentation-based tracking.

## 3. Method

### 3.1. Overall Framework

**Figure 2:**
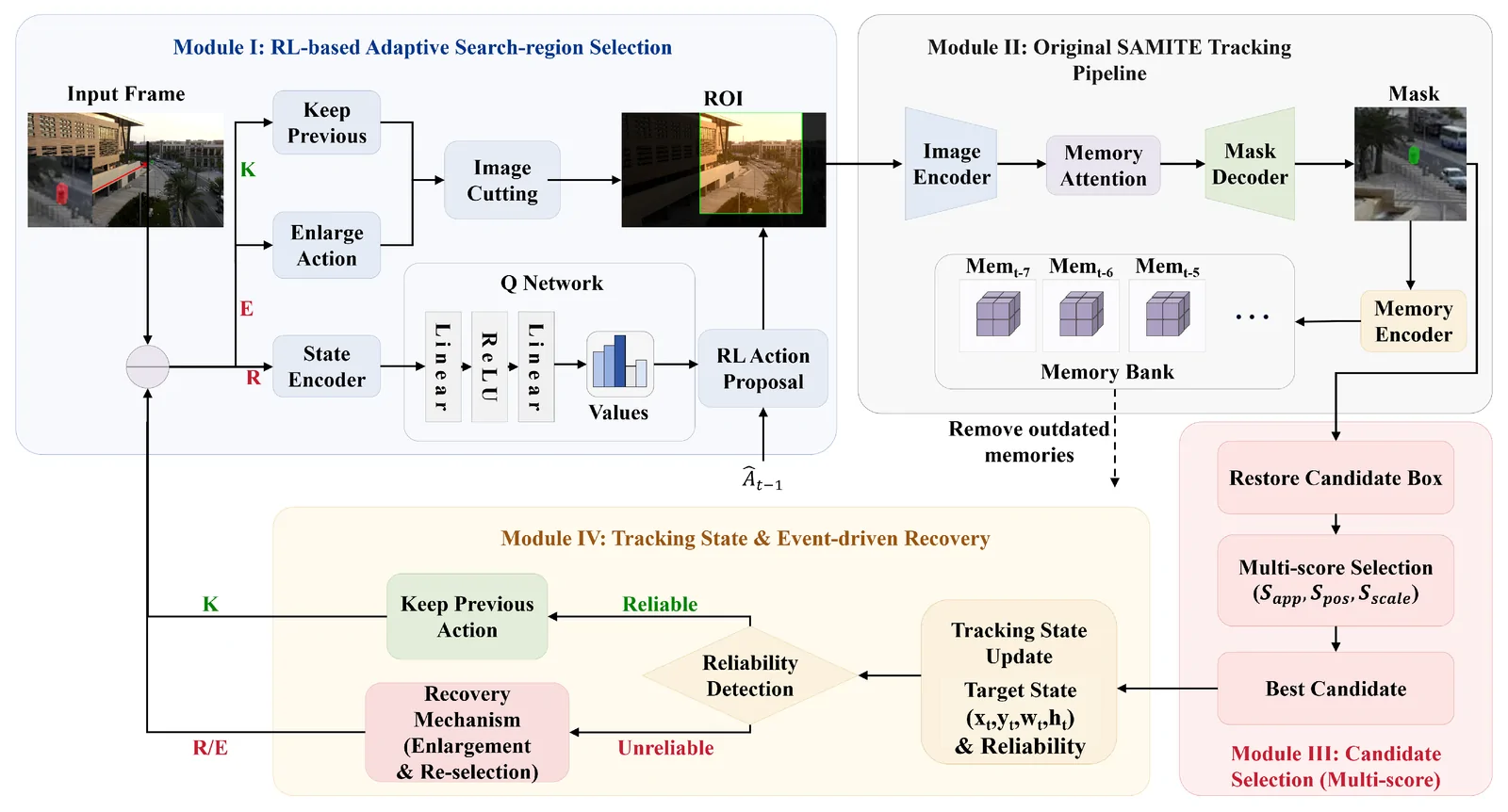
Overview of the proposed AdaSAM2 framework. The framework consists of four functional modules: (1) **RL-Based Adaptive Search-Region Selection:** the current tracking state is encoded by the Q-network to select a candidate cropping action, while the event-driven recovery mechanism determines whether to keep the previous action, progressively enlarge the search region, or invoke policy re-selection; (2) **SAMITE Tracking Pipeline:** the selected ROI is processed by the image encoder, memory attention, and mask decoder, with historical features maintained in the memory bank; (3) **Candidate Selection:** multiple candidate predictions are evaluated using appearance, spatial, and scale consistency to obtain the best target candidate; (4) **Tracking State and Event-Driven Recovery:** the selected target state and tracking reliability are updated to determine whether the previous cropping action can be retained or a recovery operation is required.

We propose AdaSAM2, an adaptive input-space optimization framework built upon SAMITE to address the resolution–context trade-off in segmentation-based object tracking. As illustrated in Figure 2, AdaSAM2 consists of four functional modules: RL-based adaptive search-region selection, the SAMITE tracking pipeline, multi-score candidate selection, and tracking-state-based event-driven recovery. The adaptive search-region module determines the cropping action according to the current tracking state, while the SAMITE pipeline performs visual feature extraction, memory-based segmentation, and mask prediction within the selected region. The candidate selection module identifies the most reliable target prediction, and the tracking-state module evaluates the resulting tracking reliability to determine whether the current cropping action should be retained or a recovery operation should be performed.

The four modules form an event-driven tracking loop rather than a strictly one-pass pipeline. First, the RL-based Adaptive Search-region Selection module determines the search-region configuration according to the current tracking state. During reliable tracking, the previously selected configuration is retained, whereas unreliable tracking activates the recovery mechanism. Second, the selected ROI is processed by the SAMITE Tracking Pipeline, which consists of image encoding, memory attention, and mask decoding, with historical features maintained in the memory bank. Third, the Candidate Selection module evaluates the resulting candidate predictions using appearance, spatial, and scale consistency and selects the most reliable target candidate. Finally, the Tracking State and Event-driven Recovery module updates the target state and tracking reliability. If the tracking state remains reliable, the previous cropping action is retained; otherwise, the recovery mechanism progressively enlarges the search region and, when the predefined recovery condition is satisfied, invokes the RL policy to propose a new candidate action. The resulting decision is then fed back to the adaptive cropping module for subsequent frames.

Among these modules, the SAMITE tracking pipeline and its associated memory mechanism are inherited from the underlying tracker, whereas adaptive search-region selection, multi-score candidate evaluation, and the event-driven tracking-state recovery mechanism constitute the main modifications introduced by AdaSAM2. During reliable tracking, the previously selected cropping configuration is reused without additional policy inference. When unreliable tracking is detected, the recovery mechanism first progressively enlarges the search region. If the unreliable state persists until the predefined recovery condition, the RL policy is invoked to propose a new candidate cropping action. The candidate action is then constrained by the previous reliable configuration before being adopted. This event-driven design enables AdaSAM2 to adapt the input space when necessary while avoiding redundant policy inference during most tracking frames.

### 3.2. SAMITE-Based Tracking Pipeline

We retain SAMITE as the underlying segmentation-based tracker of AdaSAM2. SAMITE provides the feature encoding, memory-augmented segmentation, and target prediction components, while AdaSAM2 focuses on adaptively determining the search region before these operations. This design allows the proposed search-region optimization strategy to be evaluated within a consistent segmentation tracking pipeline while preserving the original SAMITE tracking components.

Given the search region determined by the adaptive cropping module, each frame is processed within the corresponding cropped ROI. SAMITE encodes the ROI and generates candidate target masks using its memory-augmented segmentation mechanism. To maintain an efficient memory representation, outdated memory entries are pruned under a fixed budget. The resulting candidate masks are then evaluated using appearance, spatial, and scale cues, and the candidate with the highest unified score is selected as the current target prediction.

For candidate selection, we define the unified score as

(1) $$\mathrm{Score}=\alpha S_{\mathrm{app}}+\beta S_{\mathrm{pos}}+\lambda S_{\mathrm{scale}},$$

where α, β, and λ are weighting coefficients for the appearance, position, and scale cues, respectively. These three cues provide complementary information for reliable target estimation. Appearance similarity measures the semantic consistency between the current candidate and the reference target, spatial consistency suppresses abrupt localization changes, and scale consistency penalizes unreasonable variations in target size. Their weighted combination provides a more robust criterion than relying on a single cue.

The appearance similarity is computed as the cosine similarity between the current candidate and the reference target:

(2) $$S_{\mathrm{app}}=\frac{f_{t}\cdot f_{\mathrm{ref}}}{∥f_{t}∥∥f_{\mathrm{ref}}∥},$$

where $f_{t}$ and $f_{\mathrm{ref}}$ denote the feature embeddings of the current candidate and the reference target, respectively.

The spatial consistency score is defined as

(3) $$S_{\mathrm{pos}}=\exp\left(-\frac{∥c_{t}-c_{\mathrm{ref}}∥}{\sigma }\right),$$

where $c_{t}$ and $c_{\mathrm{ref}}$ denote the center coordinates of the current candidate and the reference target, respectively, and σ controls the sensitivity to spatial displacement.

The scale consistency score is defined as

(4) $$S_{\mathrm{scale}}=\frac{1}{2}\left[\exp\left(-\left|\log\frac{w_{t}}{w_{\mathrm{ref}}}\right|\right)+\exp\left(-\left|\log\frac{h_{t}}{h_{\mathrm{ref}}}\right|\right)\right],$$

where $\left(w_{t},h_{t}\right)$ and $\left(w_{\mathrm{ref}},h_{\mathrm{ref}}\right)$ denote the widths and heights of the current candidate and the reference target, respectively.

### 3.3. RL-Based Adaptive Search-Region Optimization

To adapt the input search region to the current target scale, we formulate search-region selection as a sequential decision-making problem and employ reinforcement learning (RL) to determine an appropriate cropping scale. The objective is to balance two competing requirements: a sufficiently small search region provides higher effective target resolution, whereas a larger region preserves more contextual information and improves robustness under target motion and appearance changes.

Unlike conventional approaches that perform action prediction on every frame, AdaSAM2 adopts an event-driven policy execution strategy. The RL policy is activated only at initialization or when the current tracking state becomes unreliable. During stable tracking, the previously selected cropping configuration is directly reused. This design separates search-region decision making from frame-by-frame target segmentation and avoids redundant policy inference during stable tracking.

The adaptive search-region optimization consists of three stages. First, the target geometry is converted into a compact state representation that describes the resolution requirement of the current target. Second, the RL policy selects a discrete cropping scale from a predefined action space. Third, the selected search region is maintained during reliable tracking and updated through a lost-aware recovery mechanism when tracking becomes unreliable. The Markov decision process, state and action representations, and event-driven execution strategy are described below.

#### 3.3.1. MDP Formulation

We formulate adaptive search-region selection as a Markov Decision Process (MDP),

(5) M=(S,A,P,R,γ),

where *S* denotes the state space, *A* the action space, *P* the state transition probability, *R* the reward function, and γ the discount factor. At each decision step, the agent observes the current target state $s_{t}\in S$, selects a cropping action $a_{t}\in A$, and receives a reward according to the resulting tracking quality. The subsequent target state is determined by the tracking process and is used for the next policy decision.

#### 3.3.2. State Representation

The state is constructed from the normalized geometric representation of the predicted target bounding box, which directly reflects the resolution requirement of the input search region:

(6) $$s_{t}=\left[\log\left(\frac{w_{t}}{W}\right),\log\left(\frac{h_{t}}{H}\right),\log\left(\frac{w_{t}}{h_{t}}\right),\sqrt{\frac{w_{t}h_{t}}{WH}}\right],$$

where $w_{t}$ and $h_{t}$ denote the width and height of the predicted target bounding box at time *t*, while *W* and *H* denote the width and height of the image frame, respectively. The state therefore encodes the normalized target scale, aspect ratio, and relative target area.

We intentionally use a compact geometric representation rather than incorporating additional tracking-related cues. The purpose of the RL module is to determine the appropriate input search region rather than to replace target localization or motion estimation performed by the underlying SAM2-based tracker. Geometric target size and aspect ratio directly determine the effective resolution required by the cropped input and therefore provide sufficient information for the search-region decision. In particular, fast target motion is not explicitly modeled as a velocity variable in the RL state. Instead, its impact on the validity of the current search region is handled by the lost-aware recovery mechanism, which progressively enlarges the search region and triggers policy re-selection when the tracking state remains unreliable. Additional features such as motion velocity, confidence scores, or background complexity may provide complementary information, but incorporating them would increase the state dimensionality while partially overlapping with information already modeled by the underlying tracker.

#### 3.3.3. Action Space

The action space consists of discrete cropping scales rather than continuous geometric transformations:

(7) $$a_{t}\in \left\{a_{0},a_{1},a_{2},a_{3},a_{4}\right\},$$

where $a_{t}$ denotes the selected action at time *t*, and each action corresponds to a predefined scaling factor for adjusting the search-region size.

Although a continuous action space provides a theoretically larger optimization space, it may introduce unnecessary exploration complexity for adaptive cropping. Small variations in crop boundaries often produce similar effective target resolutions and contextual information, resulting in redundant actions. We therefore adopt several representative discrete cropping scales to reduce exploration complexity while retaining sufficient flexibility to adjust the resolution–context trade-off.

The cropping region is centered according to the predicted target location, while the cropping scale is treated as the primary control variable. This design focuses the RL policy on input-resolution optimization without introducing additional object-shape or motion-estimation variables that are already handled by the underlying SAM2-based tracker.

#### 3.3.4. Event-Driven Policy Execution

The RL policy is executed in an event-driven manner rather than on every frame. At initialization, the policy predicts an appropriate cropping scale according to the initial target geometry. During reliable tracking, the previously selected cropping configuration is directly reused without additional policy inference. This design avoids repeated decision making when the current search region remains suitable for the target.

When the tracking state becomes unreliable, a lost-aware recovery process is activated. The recovery process does not immediately invoke the RL policy. Instead, the search region is first progressively enlarged to provide additional contextual information and increase the probability of recovering the target. If the unreliable state persists for the predefined recovery interval, the RL policy is re-invoked to select a new candidate cropping action. Therefore, the RL policy is responsible for proposing a candidate action only when a new search-region decision is required, rather than directly determining the final cropping configuration at every frame.

#### 3.3.5. Lost-Aware Recovery

When unreliable tracking is detected, the lost-aware recovery mechanism is activated to increase the probability of re-observing the target. During this process, the previously selected cropping configuration is retained as long as tracking remains reliable. Once tracking becomes unreliable, the recovery procedure progressively enlarges the search region according to the number of consecutive unreliable tracking events. If the unreliable state persists until the predefined recovery interval, the RL policy is re-invoked to select a new candidate cropping action.

The RL output is not directly applied as the final cropping configuration. Instead, the newly selected action is compared with the last reliable cropping configuration. If the resulting change is excessively large, the candidate action is rejected and the previous reliable configuration is retained, since an abrupt change in input resolution may further destabilize tracking. Otherwise, the new configuration is adopted. Once reliable tracking is restored, the selected configuration is reused until another unreliable state is detected. Thus, the RL policy provides the candidate action, whereas the lost-aware recovery rules determine when re-selection is required and whether the candidate action is safe to apply.

#### 3.3.6. Reward Design

The reward function is designed to encourage search-region selections that improve tracking accuracy while maintaining stable cropping behavior. It jointly considers localization quality, temporal improvement, tracking reliability, action stability, and contextual preservation:

(8) $$r_{t}=r_{\mathrm{IoU}}+r_{\mathrm{smooth}}+r_{\mathrm{th}}+r_{\mathrm{cost}}+r_{\mathrm{big}}.$$

The IoU reward provides the primary localization signal:

(9) $$r_{\mathrm{IoU}}=\mathrm{IoU}\left(B_{t},B_{t}^{gt}\right),$$

where $B_{t}$ and $B_{t}^{gt}$ denote the predicted and ground-truth bounding boxes at time *t*, respectively.

To encourage stable improvements in tracking quality, we introduce a temporal smoothness term:

(10) $$r_{\mathrm{smooth}}=\left\{\begin{array}{cc} \lambda_{s}\Delta_{t},\; & 0<\Delta_{t}<\delta ,\\ 0,\; & \mathrm{otherwise}, \end{array}\right.$$

where

(11) $$\Delta_{t}={\mathrm{IoU}}_{t}-{\mathrm{IoU}}_{t-1}.$$

This term provides additional positive feedback when the tracking quality improves moderately between consecutive steps, while avoiding excessive reward variations caused by abrupt changes. In our implementation, $\lambda_{s}=0.3$ and δ=0.1.

A threshold-based term is introduced to provide additional guidance for reliable and unreliable tracking states:

(12) $$r_{\mathrm{th}}=\lambda_{h}\max\left({\mathrm{IoU}}_{t}-\tau_{h},0\right)-\lambda_{l}\max\left(\tau_{l}-{\mathrm{IoU}}_{t},0\right),$$

where $\tau_{h}$ and $\tau_{l}$ denote the high and low IoU thresholds, respectively. The first term provides additional positive feedback for reliable predictions, whereas the second penalizes poor localization. We set $\lambda_{h}=0.3$, $\lambda_{l}=0.5$, $\tau_{h}=0.6$, and $\tau_{l}=0.3$.

To discourage unnecessarily aggressive cropping and frequent changes in the search-region scale, an action-dependent cost is introduced:

(13) $$r_{\mathrm{cost}}=c\left(a_{t}\right)-\lambda_{c}I\left(a_{t}\neq a_{t-1}\right),$$

where $c(a_{t})$ denotes the action-dependent penalty and I(·) is the indicator function. For the five discrete actions, the corresponding penalties are

(14) $$c\left(a_{t}\right)\in \left\{0,-0.05,-0.10,-0.15,-0.20\right\},$$

respectively. In addition, $\lambda_{c}=0.05$ is used to impose a small penalty when the selected action changes from the previous step. This term encourages stable search-region selection while retaining the flexibility to select smaller regions when they provide sufficient tracking benefits.

Finally, a contextual-preservation term is introduced for relatively large and reliably tracked targets:

(15) $$r_{\mathrm{big}}=\left\{\begin{array}{cc} \lambda_{b},\; & \rho_{t}>\tau_{s},\;a_{t}=0,\;{\mathrm{IoU}}_{t}>\tau_{q},\\ 0,\; & \mathrm{otherwise}, \end{array}\right.$$

where

(16) $$\rho_{t}=\frac{w_{t}h_{t}}{WH}$$

denotes the ratio between the predicted target area and the image area. When the target occupies a sufficiently large portion of the image and the tracking result is reliable, this term rewards retaining the full image rather than unnecessarily reducing the contextual information. In our implementation, $\lambda_{b}=0.2$, $\tau_{s}=0.001$, and $\tau_{q}=0.5$.

The resulting reward function combines localization accuracy, temporal improvement, tracking reliability, action stability, and contextual preservation, guiding the policy to balance effective target resolution and contextual information.The same reward formulation and parameter settings are used across all evaluation datasets without dataset-specific tuning of the reward weights or thresholds. This fixed configuration ensures that the evaluation reflects the generalization of the learned policy and recovery mechanism rather than dataset-specific reward engineering.

### 3.4. Training Strategy

#### 3.4.1. RL Architecture

The cropping policy is modeled by a lightweight Q-network implemented as a two-layer multilayer perceptron (MLP) with a hidden dimension of 128 and ReLU activation. Since the proposed state contains only four geometric variables, a lightweight MLP is sufficient to approximate the action-value function while introducing negligible computational overhead. Given the state representation $s_{t}$, the network outputs the Q-values of all candidate cropping actions:

(17) $$Q(s_{t},a;\theta ),\;a\in A,$$

where θ denotes the parameters of the online Q-network. The action with the highest Q-value is selected according to the learned policy during inference.

#### 3.4.2. Training and Inference

During training, the RL agent interacts with the tracking environment in a step-wise manner, allowing it to explore different cropping actions and learn their long-term effects on tracking performance. The action-value function is optimized using transitions collected from the training environment.

During inference, the learned policy follows the event-driven execution strategy. Specifically, the policy is invoked during initialization and unreliable tracking states, whereas the previously selected cropping configuration is reused during reliable tracking. Therefore, the training process provides sufficient exploration for learning the action-value function, while the event-driven inference strategy avoids redundant policy execution during stable tracking.

#### 3.4.3. Double DQN Optimization

The Q-network is trained using Double DQN [14]. The objective is to maximize the expected discounted cumulative reward:

(18) $$\max_{\pi }\;E\left[\sum_{t}\gamma^{t}r_{t}\right].$$

For a transition $\left(s,a,r,s^{\prime }\right)$, the Q-network is optimized using the following loss:

(19) $$L=E_{\left(s,a,r,s^{\prime }\right)}\left[{\left(Q(s,a;\theta )-y\right)}^{2}\right],$$

where the target value is computed as

(20) $$y=r+\gamma Q\left(s^{\prime },\arg\max_{a^{\prime }}Q\left(s^{\prime },a^{\prime };\theta \right);\theta^{-}\right),$$

where θ and $\theta^{-}$ denote the parameters of the online and target networks, respectively. The online network is used to select the next action, whereas the target network evaluates the selected action. The target network is periodically updated from the online network to improve training stability and reduce the overestimation bias associated with conventional Q-learning.

## 4. Experiment

### 4.1. Implementation Details

Our method is implemented based on the publicly available SAMITE framework, while all remaining settings follow the original implementation unless otherwise specified.

**Training Settings.** We use a replay buffer of size 10,000 and a batch size of 128. The learning rate is $3\times 10^{-4}$ with a discount factor of 0.95, and the target network is updated every 10 iterations. The model is optimized using Adam.

An ϵ-greedy strategy is adopted, where ϵ decays from 0.6 to 0.05 during training.

The reward-related thresholds are empirically selected to balance localization accuracy and policy stability. Specifically, $\tau_{h}=0.6$, $\tau_{l}=0.3$, $\tau_{s}=0.001$, and $\tau_{q}=0.5$.

**Action Space.** The action space consists of five discrete cropping scales defined with respect to the longer side of the image:

$$\{0,\frac{1}{1.5},\frac{1}{2},\frac{1}{3},\frac{1}{4}\}.$$

Let L=max(W,H) denote the longer side of the input image. Here, 0 denotes using the entire image, while the remaining actions correspond to square search regions with side lengths of L/1.5, L/2, L/3, and L/4, respectively. Each search region is centered at the predicted target location.

**Training Strategy.** During training, the cropping action for the first frame is randomly initialized, and the corresponding transitions are excluded from the replay buffer. This avoids introducing unreliable supervision from unstable initial states and improves policy robustness.

**Lost-aware Inference.** During inference, the policy is applied in an event-driven manner. The cropping scale is determined at initialization and reused during stable tracking to improve efficiency. Once tracking failure is detected, a lost-aware recovery strategy is activated, where the system alternates between enlarging the search region and re-selecting actions from the policy at a fixed temporal interval (every 10 frames).

**Evaluation Metrics.** We evaluate tracking performance using the success and precision metrics. For each frame, the overlap between the predicted bounding box $B_{t}$ and the ground-truth bounding box $B_{t}^{gt}$ is measured by the intersection over union (IoU):

(21) $${\mathrm{IoU}}_{t}=\frac{|B_{t}\cap B_{t}^{gt}|}{|B_{t}\cup B_{t}^{gt}|}.$$

For the success metric, the success rate at an overlap threshold τ is defined as

(22) $$S\left(\tau \right)=\frac{1}{N}\sum_{t=1}^{N}I\left({\mathrm{IoU}}_{t}>\tau \right),$$

where *N* denotes the number of valid frames and I(·) is the indicator function. The success score is calculated from the success plot by averaging the success rates over uniformly sampled overlap thresholds from 0 to 1 with an interval of 0.05.

For the precision metric, we first calculate the Euclidean distance between the centers of the predicted and ground-truth bounding boxes:

(23) $$d_{t}={∥c_{t}-c_{t}^{gt}∥}_{2}.$$

The precision at a distance threshold δ is defined as

(24) $$P\left(\delta \right)=\frac{1}{N}\sum_{t=1}^{N}I\left(d_{t}\leq \delta \right).$$

Following the evaluation protocol used in our experiments, we report Precision@20, corresponding to a 20-pixel center-error threshold. We additionally report normalized precision, where the center localization error is normalized by the ground-truth target dimensions, and use a threshold of 0.2.

### 4.2. Efficiency and Computational Overhead Analysis

The proposed RL policy follows an event-driven execution strategy and is activated only during initialization and unreliable tracking states, rather than being executed for every frame. Since the underlying SAMITE tracker remains unchanged, we focus on the additional computational overhead introduced by the proposed adaptive cropping policy. To this end, we record the policy invocation frequency and inference time on the TSFMO benchmark.

As shown in Table 1, among 50,679 processed frames, the RL policy is invoked 374 times in total. Among these invocations, 260 occur during initialization and 114 are triggered during the subsequent recovery process. Thus, only 0.74% of all processed frames require an RL invocation, while recovery-triggered invocations account for only 0.23% of all frames. The accumulated inference time of the RL module is only 0.43 s, resulting in an average inference time of 1.15 ms per policy invocation and an average additional overhead of only 0.0085 ms per frame.

These results demonstrate that the event-driven policy introduces only a negligible additional computational cost while retaining the ability to adapt the search region when necessary. The low invocation frequency also indicates that the selected cropping configuration can be reused for most tracking frames. After initialization, policy re-selection is triggered only during challenging tracking periods when progressive search-region enlargement alone is insufficient and a new action decision is required. This behavior is consistent with the design objective of the event-driven strategy, which allows the RL policy to intervene only when necessary rather than performing per-frame action prediction.

### 4.3. Tracking Performance on Small and General UAV Benchmarks

#### 4.3.1. Performance on Small-Object Benchmarks

We first evaluate AdaSAM2 on TSFMO [8] and LaTOT [9], which contain challenging small object tracking scenarios characterized by small target sizes, rapid motion, and substantial appearance variation. Table 2 reports the quantitative comparison with representative state-of-the-art trackers.

AdaSAM2 consistently improves upon the SAMITE baseline on both benchmarks. On TSFMO, AdaSAM2 achieves 43.9% Success, 63.0% normalized precision, and 75.3% Precision, improving over SAMITE by 1.3, 2.5, and 1.3 percentage points, respectively. On LaTOT, AdaSAM2 achieves 38.6% Success, 53.4% normalized precision, and 74.3% Precision, corresponding to improvements of 0.8, 2.8, and 2.5 percentage points, respectively. Among the compared methods, AdaSAM2 achieves the best overall performance on both small-object benchmarks.

**Figure 3:**
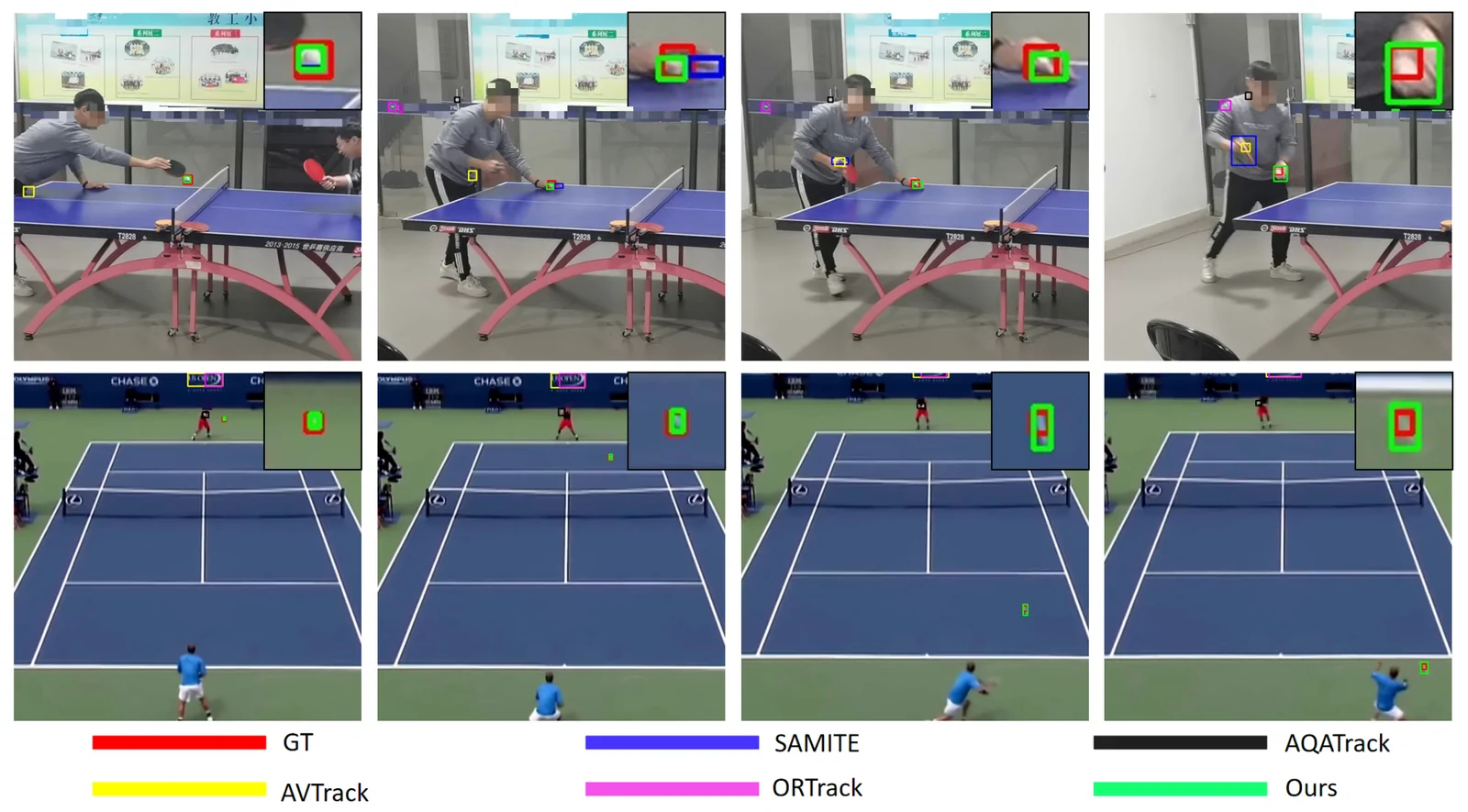
Qualitative comparison of different trackers on representative object tracking sequences. The ground-truth annotations are shown together with the predictions of competing trackers and AdaSAM2.

**Qualitative Analysis.** Figure 3 presents qualitative comparisons between AdaSAM2, competing trackers, and the ground truth under representative object tracking scenarios. AdaSAM2 maintains more accurate target localization and exhibits reduced drift compared with the competing methods. These results qualitatively support the effectiveness of adaptive search-region optimization in improving target representation while retaining sufficient contextual information.

#### 4.3.2. Generalization Across Target Scales

To further examine whether the learned adaptive cropping policy is restricted to small-object scenarios, we evaluate AdaSAM2 on UAV123 [1], UAV123@10FPS [1], and UAVDT [2]. These benchmarks contain more general UAV tracking scenarios with broader variations in target scale, motion patterns, and temporal sampling conditions. The results are summarized in Table 3.

AdaSAM2 consistently outperforms the SAMITE baseline across all three benchmarks. On UAV123, AdaSAM2 improves Success and Precision from 69.6% and 92.7% to 71.1% and 94.8%, respectively. On UAV123@10FPS, the corresponding improvements are from 70.5% and 92.9% to 71.3% and 94.2%. On UAVDT, AdaSAM2 further improves Success and Precision from 71.0% and 95.8% to 72.1% and 96.7%. The consistent gains across these datasets indicate that the proposed adaptive search-region strategy remains effective under different target scales and temporal sampling conditions.

**Cross-Scale Analysis.** The results demonstrate that the proposed RL-based adaptive cropping strategy can adjust the input search region according to the target geometry. For relatively small targets, tighter search regions can increase the effective target resolution, whereas larger regions can preserve contextual information when the target occupies a larger portion of the frame or when recovery is required. This adaptive behavior enables the proposed method to maintain consistent improvements across datasets with different target-scale distributions, suggesting that the learned policy is not limited to small-object tracking scenarios.

### 4.4. Ablation Study

#### 4.4.1. Component Ablation

To investigate the contribution of the proposed recovery components, we perform an ablation study on UAV123 [1] and TSFMO [8]. Specifically, we evaluate two components: the constrained policy re-selection mechanism and the progressive search-region enlargement strategy. The results are reported in Table 4.

The baseline configuration without either recovery component achieves 69.6% Success and 92.7% Precision on UAV123, and 42.6% Success and 74.0% Precision on TSFMO. Introducing the re-selection constraint improves Success and Precision to 70.6% and 94.0% on UAV123, respectively, while increasing the corresponding values on TSFMO to 43.3% and 74.3%. These results indicate that constraining policy re-selection helps prevent unstable changes in the search region and therefore improves tracking stability.

When only the progressive enlargement strategy is enabled, Success increases to 70.5% on UAV123 and 42.9% on TSFMO. Although the Precision on TSFMO decreases slightly, the improvement in Success indicates that progressive enlargement provides additional recovery capability when the target becomes difficult to localize.

When both components are enabled, AdaSAM2 achieves the best performance on both datasets, reaching 71.1% Success and 94.8% Precision on UAV123, and 43.9% Success and 75.3% Precision on TSFMO. The combined improvement is larger than that obtained by either component alone, demonstrating that constrained policy re-selection and progressive search-region enlargement play complementary roles in the recovery process.It should be noted that the RL-based action selection is not an independent recovery component in this framework. The RL policy is conditionally invoked as part of the lost-aware recovery process, and its output is subsequently subject to the constrained re-decision rule. Removing the RL selector alone would require replacing the re-selection step with another action-selection strategy, thereby changing the recovery policy rather than isolating a single component. The contribution of learned action selection is therefore further examined in Table 5 by comparing the RL-based strategy with manually designed fixed cropping policies.

#### 4.4.2. Comparison with Fixed Cropping Strategies

To further verify the necessity of adaptive policy learning, we compare the proposed RL-based cropping strategy with manually designed fixed cropping policies. For a fair comparison, all fixed-policy variants use the same underlying tracker and lost-aware recovery framework as the proposed method. The only difference is the search-region selection strategy: the proposed method uses the learned RL policy, whereas the heuristic variants use manually specified thresholds and crop scales. Specifically, the fixed policies determine the crop scale according to the target-to-image area ratio, using two thresholds (0.0005 and 0.001) and two representative crop scales (1/2 and 1/3). The results are summarized in Table 5.

The results show that fixed cropping policies can provide improvements under specific target-scale distributions, but their effectiveness is highly dependent on the selected threshold and crop scale. For example, the 1/2 cropping strategy with a threshold of 0.0005 achieves the best fixed-policy performance on UAV123, improving Success and Precision by 1.3 and 2.0 percentage points over SAMITE, respectively. However, the same strategy provides only a marginal Success improvement on TSFMO and results in a Precision decrease.

More aggressive cropping with a scale of 1/3 further illustrates the limitation of fixed policies. Although it improves Precision on UAV123 relative to the SAMITE baseline, it substantially degrades both Success and Precision on TSFMO. This suggests that a fixed crop scale cannot simultaneously accommodate datasets with different target-scale and motion characteristics.

We further note that the competitive performance of the best heuristic on UAV123 does not diminish the value of adaptive policy learning. Instead, it reflects the scale distribution of the UAV123 dataset, which contains relatively few extremely small targets. For scenarios dominated by small-to-medium sized objects, a carefully hand-tuned fixed crop scale can serve as a strong baseline. However, its effectiveness is brittle: the same heuristic that works well on UAV123 fails severely on TSFMO, a benchmark characterized by tiny and fast-moving targets. In contrast, the proposed RL-based policy consistently improves performance across both datasets without requiring manual re-tuning, demonstrating superior generalization. For completeness, we emphasize that all heuristic variants use the exact same lost-aware recovery framework (progressive enlargement and constrained re-decision) as the proposed method; the only difference is the crop-scale selection strategy, i.e., learned RL policy versus manually specified rules. Therefore, the comparison cleanly isolates the benefit of adaptive policy learning.

In contrast, the proposed RL-based policy does not rely on a manually specified crop scale for different target conditions. Instead, it learns to select the search-region scale according to the target geometry and dynamically adjusts the cropping strategy during tracking and recovery. Therefore, the comparison demonstrates the advantage of adaptive policy learning over manually designed fixed cropping rules, rather than simply showing that a particular crop scale is superior.

### 4.5. Sensitivity Analysis of the Recovery Interval

The proposed lost-aware recovery mechanism alternates between progressive search-region enlargement and RL-based policy re-selection. The recovery interval determines the number of consecutive unreliable tracking events before the next recovery decision is performed and therefore affects the trade-off between recovery responsiveness and policy stability. To investigate this effect, we evaluate three interval settings, i.e., 5, 10, and 20 frames, on TSFMO and UAVDT. The results are reported in Table 6.

The results show that the three recovery intervals lead to relatively similar performance, indicating that the proposed recovery mechanism is not highly sensitive to the interval within the tested range. An interval of 10 frames achieves the best overall results across the two datasets, although the differences from the 5-frame setting are small, particularly on TSFMO. When the interval is reduced to 5 frames, more frequent recovery decisions may introduce unnecessary changes in the search region before sufficient unreliable-tracking evidence is accumulated.

In contrast, increasing the interval to 20 frames delays policy re-selection after tracking failure. Although the progressive search-region enlargement provides an additional recovery mechanism, the tracker may remain in a suboptimal cropping configuration for a longer period, resulting in slightly lower performance.

Overall, the results indicate that the recovery interval provides a trade-off between recovery responsiveness and policy stability. The 10-frame interval yields the best overall performance across the two datasets and is therefore adopted as the default setting throughout the experiments. Nevertheless, the relatively small performance differences among the tested settings indicate that the proposed recovery mechanism is relatively insensitive to the exact choice of interval.

## 5. Discussion

The experimental results demonstrate that adaptive input-space optimization effectively mitigates the resolution–context trade-off that fundamentally limits small-object tracking. Unlike existing SAM2-based trackers that rely on fixed or heuristic search regions, AdaSAM2 dynamically adjusts the cropping scale according to the available geometric tracking state, improving the effective resolution of tiny targets while preserving sufficient contextual information. This is particularly evident on TSFMO, where the relative gain over SAMITE (+1.3% Success) is associated with the adaptive selection of search-region scales in challenging sequences, as qualitatively shown in Figure 3. Although some of these sequences contain fast motion and motion blur, these cues are not explicitly included in the RL state. Therefore, this result should not be interpreted as evidence that the RL policy directly detects fast motion or blur. Instead, the policy selects the search-region scale from the available geometric tracking state, while the lost-aware recovery mechanism provides additional robustness when the current search region becomes unreliable.

Our results further reveal an important insight: many tracking failures in SAM2-based methods stem not only from model capacity, but also from suboptimal input representation. This finding aligns with recent observations in the small-object tracking literature [10] and suggests that adaptive input control offers a complementary direction to architectural improvements for segmentation-based tracking. The consistent improvements across both small-object (TSFMO, LaTOT) and general (UAV123, UAVDT) benchmarks further indicate that the learned policy generalizes across different target scales rather than being restricted to tiny-object scenarios.

The event-driven design is central to the practicality of AdaSAM2. By invoking the RL policy only during initialization and when the lost-aware recovery process requires a new action decision, our method introduces negligible per-frame overhead (0.0085 ms on average). During reliable tracking, the previously selected cropping configuration is reused without additional policy inference. When tracking becomes unreliable, the recovery mechanism first progressively enlarges the search region according to the number of consecutive unreliable tracking events. If the unreliable state persists until the predefined recovery interval, the RL policy is re-invoked to propose a new candidate cropping action. The candidate action is subsequently subject to the constrained re-decision rule: excessively large changes relative to the last reliable configuration are rejected, while reasonable actions are adopted. This design allows the RL policy to provide adaptive candidate actions without directly determining the final cropping configuration or introducing abrupt changes in the input resolution.

While the proposed framework demonstrates strong empirical performance, several limitations warrant future investigation. First, the current RL policy is trained offline and remains fixed during inference; exploring online adaptation or meta-learning strategies could further improve generalization across unseen scenarios. Second, the state representation is intentionally limited to geometric features to avoid redundancy with the visual tracking information already modeled by the underlying SAM2-based tracker. Although the current lost-aware recovery mechanism provides robustness to unreliable tracking caused by challenging motion, incorporating lightweight motion cues may provide additional benefits for extremely fast-moving targets and is therefore an interesting direction for future work. Third, the discrete action space, though effective, may not be globally optimal; continuous action formulations with proper regularization could offer finer-grained control over the resolution–context balance.

## 6. Conclusions

In this paper, we address the resolution–context trade-off in visual tracking by treating the input search region as an adaptive component rather than a fixed preprocessing operation. We propose AdaSAM2, which formulates search-region adaptation as a sequential decision-making problem and employs reinforcement learning to select the cropping scale. The proposed event-driven policy is activated only during initialization and unreliable tracking states, while the selected configuration is reused during stable tracking. A lost-aware recovery mechanism further combines progressive search-region enlargement with constrained policy re-selection to improve robustness under target disappearance.

Extensive experiments on tracking benchmarks demonstrate the effectiveness and generalization of the proposed approach. Compared with the SAMITE baseline, AdaSAM2 improves Success and Precision from 42.6% and 74.0% to 43.9% and 75.3% on TSFMO, and from 69.6% and 92.7% to 71.1% and 94.8% on UAV123. Consistent improvements are also observed on LaTOT and UAVDT, demonstrating that the proposed adaptive search-region strategy is effective for both small-object and general UAV tracking scenarios. Moreover, the RL policy is invoked on only 0.74% of processed frames on TSFMO, with an average inference overhead of 0.0085 ms per frame, confirming the computational efficiency of the event-driven design.

Overall, AdaSAM2 provides a lightweight and plug-and-play solution for adaptive input-space optimization without retraining the core SAM2-based tracker. Future work will investigate extending the adaptive input paradigm to multi-object tracking and replacing the hand-crafted geometric state with learned visual representations to better capture background clutter, motion blur, and other contextual cues.

## Figures and Tables

**Table 1:** Computational overhead of the proposed RL policy on TSFMO.

Metric	Value
Processed frames	50,679
Initialization invocations	260
Recovery-triggered invocations	114
Total RL invocations	374
Invocation ratio	0.74%
Total RL inference time	0.43 s
Average time per invocation	1.15 ms
Average overhead per frame	0.0085 ms

**Table 2:** Performance comparison with state-of-the-art trackers on TSFMO and LaTOT benchmarks. The best, second-best, and third-best results are highlighted in red, blue, and green, respectively.

Method	Source	TSFMO			LaTOT		
		Succ.	Norm.Prec	Prec.	Succ.	Norm.Prec	Prec.
OSTrack	ECCV22	21.9	33.1	31.1	–	–	–
S-KeepTrack	ACCV22	25.5	**42.5**	**43.7**	–	–	–
DropTrack	CVPR23	23.7	35.6	35.7	32.8	36.5	56.9
AVTrack	ICML24	19.2	30.4	27.3	27.0	31.7	48.1
AQATrack	CVPR24	27.8	40.3	40.0	33.0	36.9	**57.2**
HIPTrack	CVPR24	26.7	39.2	38.4	33.0	36.9	56.7
MKDNet	IJCV24	–	–	–	**34.0**	**41.3**	53.9
ORTrack	CVPR25	20.4	33.1	37.8	27.2	31.7	48.2
SUTrack	AAAI25	**28.7**	41.2	41.2	–	–	–
SAMITE	arXiv25	**42.6**	**60.5**	**74.0**	**37.8**	**50.6**	**71.8**
AdaSAM2	Ours	**43.9**	**63.0**	**75.3**	**38.6**	**53.4**	**74.3**

**Table 3:** Comparison with state-of-the-art trackers on UAV123 [1], UAV123@10FPS [1], and UAVDT [2]. Performance is evaluated in terms of success (Succ.) and precision (Prec.). The best, second-best, and third-best results are highlighted in red, blue, and green, respectively.

Method	Source	UAV123		UAV123@10FPS		UAVDT	
		Succ.	Prec.	Succ.	Prec.	Succ.	Prec.
Aba-Track	ICCV23	66.4	86.4	65.5	85.0	59.9	83.4
AVTrack	ICML24	66.8	84.8	65.8	83.2	58.7	82.1
HIPTrack	CVPR24	**70.5**	89.2	**70.6**	**89.2**	60.9	79.6
ODTrack	AAAI24	**70.4**	**89.8**	70.0	88.9	**65.3**	**86.6**
SGLATrack	CVPR25	66.9	84.9	–	–	59.9	81.9
ORTrack	CVPR25	66.4	84.3	–	–	60.1	83.4
SAMITE	arXiv25	69.6	**92.7**	**70.5**	**92.9**	**71.0**	**95.8**
AdaSAM2	Ours	**71.1**	**94.8**	**71.3**	**94.2**	**72.1**	**96.7**

**Table 4:** Ablation study of the proposed components on UAV123 [1] and TSFMO [8] datasets. Best results are highlighted in bold. Here, ✓ and ✗ denote that the corresponding component is enabled and disabled, respectively.

Clamp Re-dec	Enlargement	UAV123		TSFMO	
		Succ.	Prec.	Succ.	Prec.
✗	✗	69.6	92.7	42.6	74.0
✓	✗	70.6	94.0	43.3	74.3
✗	✓	70.5	93.9	42.9	73.8
✓	✓	**71.1**	**94.8**	**43.9**	**75.3**

**Table 5:** Comparison of adaptive strategy against heuristic crop policies. Superscripts denote performance change relative to the SAMITE baseline.

Threshold	Action	UAV123 [1]		TSFMO [8]	
		Succ.	Prec.	Succ.	Prec.
0.001	1/2	$70.5^{+0.9}$	$94.4^{+1.7}$	$42.6^{0.0}$	$73.1^{-0.9}$
0.0005	1/2	$70.9^{+1.3}$	$94.7^{+2.0}$	$42.8^{+0.2}$	$73.2^{-0.8}$
0.001	1/3	$69.6^{0.0}$	$93.5^{+0.8}$	$40.3^{-2.3}$	$69.1^{-4.9}$
0.0005	1/3	$70.5^{+0.9}$	$93.8^{+1.1}$	$40.5^{-2.1}$	$69.6^{-4.4}$
Original (SAMITE)		69.6	92.7	42.6	74.0

**Table 6:** Sensitivity analysis of the recovery interval in the lost-aware recovery mechanism. The interval denotes the number of consecutive unreliable tracking events before the next recovery decision. The best results are highlighted in bold.

Interval	TSFMO		UAVDT	
	Success	Precision	Success	Precision
5	43.95	75.60	71.49	95.73
**10**	**44.01**	**75.63**	**71.80**	**96.33**
20	43.76	75.16	71.73	96.20

## Data Availability

The data presented in this study are available upon request from the corresponding author.
